# Supplementary material for: The causal relationship between the human gut microbiota and pyogenic arthritis: a Mendelian randomization study
Source: Front Cell Infect Microbiol. 2024 Nov 26;14:1452480. doi: 10.3389/fcimb.2024.1452480 (PMC11629706; doi:10.3389/fcimb.2024.1452480)
Supplement: Supplementary file 1 [file DataSheet1.zip › mendelian test/ebi-a-GCST90027549.csv_forest.pdf]

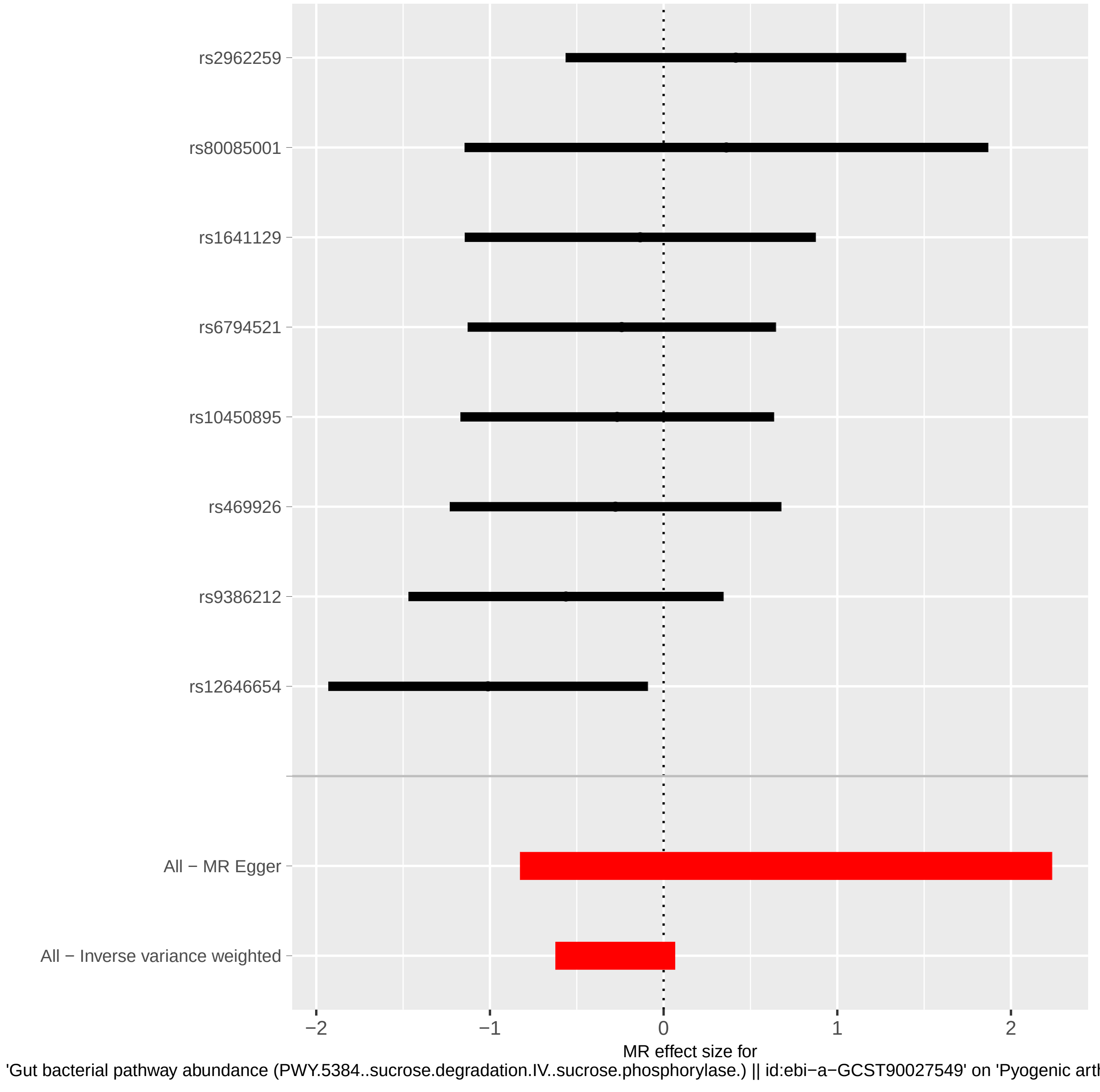

rs2962259
rs80085001
rs1641129
rs6794521
rs10450895
rs469926
rs9386212
rs12646654
All − MR Egger
All − Inverse variance weighted
−2
−1
0
1
2
MR effect size for
'Gut bacterial pathway abundance (PWY.5384..sucrose.degradation.IV..sucrose.phosphorylase.) || id:ebi−a−GCST90027549' on 'Pyogenic art
